# Book Review: *The Oxford Handbook of the Neurobiology of Pain* by J. N. Wood

**DOI:** 10.1177/03010066211053569

**Published:** 2021-10-09

**Authors:** M. Catherine Bushnell

**Affiliations:** Harold Griffith Professor Emerita, McGill University, Montreal, Quebec, Canada; Scientist Emerita, National Institutes of Health, Bethesda, MD, USA

Until recently, pain was viewed as an unpleasant symptom of disease or injury that needed to be either alleviated or tolerated, but that did not inflict harm in and of itself. We now know that pain is much more than a symptom and, when chronic, can be viewed as a disease itself. The opioid crisis, along with an aging population and the novel threat of pain related to long COVID, has drawn new light to the consequences of pain and led to the search for a better understanding of pain mechanisms and possible non-opioid treatments.

*The Oxford Handbook of the Neurobiology of Pain* is a timely offering, providing a comprehensive compendium of scholarly articles about pain, ranging from the history of pain research, through non-human models, to detailed chapters about mechanisms at peripheral and central nervous system levels, and finally pain conditions and treatment modalities. Individual chapters are dedicated to some common chronic pain syndromes, such as headache, visceral pain, neuropathic pain and cancer pain. Other chapters delve into molecular mechanisms, pain genetics, neuroimmune interactions and pain, and developmental aspects of pain.

The handbook is not organized into sections, and there is little coordination between chapters, leading to a fair amount of redundancy, particularly in terms of primary afferent and spinal cord mechanisms. This is not necessarily bad, since it allows readers to select individual chapters to read, without committing to a thorough perusal of the more than 800 pages of text. Each chapter has enough autonomy to be a stand-alone analysis of the topic and serve as a fundamental resource for scientists and trainees working in the field.

A stellar array of more than 50 experts in the field have contributed to the handbook, each presenting state-of-the art analyses of their topic. It is not possible to address each chapter individually, but some highlights include an engrossing historical overview of purported pain mechanisms and analgesic treatments throughout the ages (Chapter 1) and a thought-provoking analysis of the translation from rodent models to humans that clearly shows the problems but presents an optimistic view of the future (Chapter 2).

A number of chapters offer an excellent reference for the serious pain scientist, with detailed analyses of most aspects of nociception. Chapters are dedicated to the dorsal root ganglia (5), heat and cold pain (7), noxious mechanoreception (8), signaling channels in the periphery and dorsal horn (9-12, 21, 26, 27), and dorsal horn mechanisms (16).

The chapters on basic pain mechanisms may be too detailed for clinicians or neuroscientists not specializing in pain, but other chapters could be extremely useful to the research-minded clinician by providing a link between pre-clinical data and clinical syndromes. Such chapters include a discussion of sympathetic nervous system and pain (6); neuroimmune interactions and pain (13), developmental and sex-related aspects of pain (14), mechanisms of migraine pain (19), relationship between sleep and pain (20), the diversity of neuropathic pain conditions (22), the transition from acute to chronic (23), cancer pain (24) and opioid mechanisms (25).

Although the handbook is quite comprehensive, there are some important gaps in the topics. In terms of pre-clinical models, it was nice to see the inclusion of a chapter on invertebrate models of nociception, but a discussion of human and non-human primate studies of nociception is conspicuously absent. Additionally, although several chapters discuss descending pain modulation systems and their role in pharmacological pain modulation, there is little discussion of non-pharmacological pain treatments and their mechanisms. Given the problems with opioids and the uneven effectiveness of other pharmacological treatments for chronic pain, a deep dive into non-pharmacological pain treatments would have been welcome.

In conclusion, *The Oxford Handbook of the Neurobiology of Pain* is an outstanding collection of chapters addressing a wide range of topics in the field. With a few gaps, it is inclusive of both basic and clinical aspects of pain. The volume should appeal most to the dedicated pain scientist, but it could also be a useful tool for clinicians and students. At a price-tag of more than $200 US, and with free reviews of many topics available within scientific journals, the appeal of this book to trainees may, unfortunately, be diminished. Nevertheless, it is a definitive volume that brings together the important issues in the field of pain, discussed by the most preeminent experts.

